# A retrospective study on IVF/ICSI outcome in patients with anti-nuclear antibodies: the effects of prednisone plus low-dose aspirin adjuvant treatment

**DOI:** 10.1186/1477-7827-11-98

**Published:** 2013-10-05

**Authors:** Qing Zhu, Li Wu, Bo Xu, Mei-Hong Hu, Xian-Hong Tong, Jing-Juan Ji, Yu-Sheng Liu

**Affiliations:** 1 Centre for Reproductive Medicine, Department of Obstetrics and Gynecology, Anhui Provincial Hospital affiliated with Anhui Medical University, Hefei, Anhui 230000, China

**Keywords:** Anti-nuclear antibody, ANA, IVF/ICSI outcome, Titre, Prednisone, Aspirin

## Abstract

**Background:**

Anti-nuclear antibodies (ANA) are suspected of having relevance to adverse reproductive events.

**Methods:**

This study aims to investigate the potential effect of ANA on IVF/ICSI outcome and the therapeutic role of prednisone plus low-dose aspirin (P + A) adjuvant treatment in ANA + patients. The first IVF/ICSI cycles without P + A of sixty-six ANA + women were enrolled as the ANA + group, and the 233 first IVF/ICSI cycles of matched ANA- women served as the ANA- group. The ANA + group was divided into the Titre < =1:320 subgroup and the Titre > 1:320 subgroup. Twenty-one ANA + women with adverse outcomes in their first cycles (ANA + cycles without P + A) received P + A adjuvant treatment for three months before the second IVF/ICSI cycle (ANA + cycles with P + A). The clinical characteristics and the IVF/ICSI outcomes were compared, respectively, between 1) the ANA + group and the ANA- group, 2) the Titre < =1:320 subgroup and the Titre > 1:320 subgroup, and 3) the ANA + cycles without P + A and the ANA + cycles with P + A.

**Results:**

No significant differences were observed between each of the two-group pairs in the clinical characteristics. The ANA + group exhibited significantly lower MII oocytes rate, normal fertilisation, pregnancy and implantation rates, as well as remarkably higher abnormal fertilisation and early miscarriage rates. The Titre < =1:320 subgroup’s IVF/ICSI outcomes were as poor as those of the Titre > 1:320 subgroup. After the P + A adjuvant treatment, the number of two pro-nuclei, perfect embryos and available embryos, and the implantation rate increased significantly.

**Conclusions:**

These observations suggest that ANA could exert a detrimental effect on IVF/ICSI outcome that might not be titre-dependent, and P + A adjuvant treatment could be useful for ANA + patients. This hypothesis should be verified in further prospective randomised studies.

## Background

A portion of infertile patients consistently fail to conceive despite recurrent transfers of high-quality embryos. The mechanism leading to this reproductive failure is still unclear. To investigate this failure, several researchers have focused on the association between autoimmune factors and IVF/ICSI outcome, and the focus is especially directed toward autoantibodies [1-5]. It has been reported that ANA were relevant to adverse reproductive events, including recurrent spontaneous abortion, endometriosis, infertility, IVF failure, and ovarian dysfunction [6-10].

In a broad sense, ANA are a group of autoantibodies that target the entire cell including DNA, RNA and proteins [11]. Most of the common nuclear antigens can be found on the surface structures of apoptotic cells, and these molecules with the phospholipids on the surface of the apoptotic cells can induce an autoimmune reaction, resulting in an abnormal release of anti-phospholipids antibodies (APA) or ANA [12,13]. A basic cellular abnormality, such as increased apoptosis or decreased clearance of apoptotic cells might explain the increased APA and ANA levels in individuals with autoimmune diseases [14]. One iconoclastic theory that ANA such as an anti-double strand DNA (anti-dsDNA) antibody, anti-RNP antibody, or anti-ribosomal P protein antibody could penetrate into living cells has been confirmed *in vivo* and *in vitro*[15-17].

It has been suggested that ANA could impair oocyte quality and embryo development, leading to reduced pregnancy and implantation rates [7,10], and women with recurrent pregnancy loss had a significantly higher incidence of ANA than controls [9]. Shirota et al. [18] found that the presence of the anti-centromere antibody might interfere with the oocyte maturation from MI to MII and impair the embryo cleavage potential. Embryo development was severely impaired or failed when co-cultured with IgG from ANA + women [19].

In our study, we retrospectively analysed IVF/ICSI outcomes in ANA + women and compared them with ANA-controls. Differing from previous reports, we studied the effects of prednisone plus low-dose aspirin (P + A) adjuvant therapy in ANA + women before IVF/ICSI cycles. The aim of this study is to investigate the potential effect of ANA and different ANA titres on IVF/ICSI outcome, and to determine the role of adjuvant treatment in these ANA + patients.

## Methods

### Patients

Patients who received IVF/ICSI treatment were recruited from the Reproductive Medicine Centre of Anhui Provincial Hospital affiliated with Anhui Medical University from October 2009 to September 2012.

In our centre, ANA, D-dimmer, platelet aggregation test, anti-cardiolipin antibody (ACA), anti-beta(2)-glycoprotein I antibody (A-β2-GPI), triiodothyrorine (T3), thyroxine (T4), thyroid stimulating hormone (TSH) and anti-thyroid antibody (ATA) detection examinations were performed to exclude pre-thrombotic state and autoimmune disease. The women positive for ANA and without specific autoimmune diseases were provided with information regarding the P + A adjuvant therapy protocol, and after considering the advantages and disadvantages, they decided whether to receive the adjuvant therapy before each cycle.

A total of seventy-three patients meeting the following requirements: 1) with age not over 38 years, 2) with infertility caused by tubal disorder and/or male factor or unexplained infertility, 3) who have undergone IVF/ICSI treatment with the standard long protocol, and 4) positive for ANA and negative for other antibodies were enrolled in our study, wherein seven patients received adjuvant therapy, and the remaining sixty-six patients received no special medications before IVF/ICSI treatment. The first IVF/ICSI cycles (28 IVF cycles and 38 ICSI cycles) of these sixty-six patients were enrolled as the ANA + group.

For the controls, two hundred thirty-three patients 1) with age not over 38 years, 2) with infertility caused by tubal disorder and/or male factor, 3) who have undergone IVF/ICSI treatment with the standard long protocol in the same period as the ANA + patients, 4) negative for any antibody, and 5) who haven’t received any special medications before the IVF/ICSI treatment were recruited in our study, and only the first IVF/ICSI cycles (106 IVF cycles and 127 ICSI cycles) were enrolled as the ANA- group.

The exclusion criteria for both groups were as follows: uterine malformation; hypothyroidism; hyperthyroidism; chromosome abnormality of the couples; sexually transmitted diseases (STD) with ureaplasma urealyticum, mycoplasma, chlamydia, gonococcus, fungi, trichomonas vaginalis, HIV or treponema pallidum; or autoimmune diseases such as systemic lupus erythematosus, anti-phospholipids syndrome, autoimmune thyroiditis, and Sjogren syndrome. This study was approved by the Ethics Committee of Anhui Provincial Hospital.

According to the ANA titres, the ANA + group was divided into two subgroups, including the Titre **≤** 1:320 subgroup (46 cycles) and the Titre > 1:320 subgroup (20 cycles). The clinical characteristics and the IVF/ICSI outcomes were compared, respectively, between the ANA + group and the ANA- group, and between the two subgroups.

### Adjuvant medications

Twenty-one of these 66 ANA + patients, who had poor IVF/ICSI outcomes in their first treatment cycles, received a daily oral dose of 10 mg of prednisone plus 100 mg of aspirin up to three months before the second IVF/ICSI cycle, wherein 2 patients (9.5%) became ANA-negative, and 19 patients (90.5%) remained ANA-positive after treatment. These patients entered the IVF/ICSI program immediately after three months of adjuvant treatment and received the same long-protocol for controlled ovarian stimulation (COH) as in the first cycles. The one-paired comparison was performed between the first cycles (the ANA + cycles without P + A) and the second cycles (the ANA + cycles with P + A).

### ANA assay

The ANA were detected using the indirect immunofluorescence (IFT) method on the human epithelial (HEp-2) cell substrate. Serum samples from the patients were prepared at various dilution factors as follows: 1:100, 1:320, 1:1000, 1:3200, 1:10000, 1:32000 and incubated with fixed HEp-2 cells. The ANA could react with the antigens of the Hep-2 cell substrate, forming antigen-antibody complexes bound to the cell nucleus. Temporarily, fluorescein-labelled antihuman immunoglobulin was added and fluorescein-labelled antibody- antigen-antibody complexes were formed and detected by using fluorescence microscopy. The ANA were defined as positive when the signal could be detected in the nucleus with the serum diluted at 1:100 and the titre of the ANA correlated with the highest serum dilution factor that allowed for observing the fluorescence. The nuclear patterns were observed using the Olympus BX51 fluorescence microscope and all the antibodies kits were purchased from Euroimmune Company (Germany).

### IVF/ICSI protocol

A long pituitary down-regulation protocol was used in all the patients. A long-acting gonadotropin-releasing hormone agonist (GnRH-a, Diphereline; Ipsen Pharma Biotech, Signes, France) was injected intramuscularly in the mid-luteal phase of the preceding cycle of gonadotropin (Gn) stimulation. After the complete pituitary down-regulation, recombinant human FSH (Gonal-F, Merck Serono SA, Geneva,Switzerland) was injected for COH and urinary gonadotrophin (Menotrophins for Injection, LIVZON, China) was added in the late-follicle phase. Human chorionic gonadotropin (HCG, LIVZON, China) at a dose of 10000 IU was injected when at least two follicles had reached 18 mm in mean diameter or more than three follicles had reached 17 mm, and the serum estradiol (E2) levels indicated more than 250 pg/ml/mature follicle. The oocytes pick-up (OPU) was performed transvaginally 36 h after the HCG injection.

The selection of fertilisation program, IVF or ICSI, was based on the semen condition on the day of oocyte retrieval. The oocyte was considered to be fertilised normally if a second polar body was extruded or if two pro-nuclei (2PN) were observed 16 hours after insemination.

Embryo quality is primarily assessed by the day 3 embryo grading system in our centre. The day 3 embryos were evaluated based on the number and size of their blastomeres and the degree of fragmentation as follows: Grade 1: 6–8 even, equally sized blastomeres without fragmentation of the blastomeres; Grade 2: 6–8 even, equally sized blastomeres, and less than 20% fragmentation of the blastomeres; Grade 3: 4–6 uneven or irregularly shaped blastomeres, and 20-50% fragmentation of the blastomeres; Grade 4: the embryos are considered non-viable with more than 50% fragmentation or with even lysed, contracted or dark blastomeres. The embryos graded 1 and 2 were considered good-quality or perfect embryos, and the embryos graded 1, 2 and 3 were considered available embryos. Two to three good-quality embryos were initially selected for transfer (ET).

Progesterone supplementation by daily intramuscular injection of 40 mg/day for the initial three days and 60 mg/day for the following days (approximately three months) was administered from the day of the oocyte retrieval. Pregnancy was diagnosed by a positive blood test for β-hCG at 14 days after the embryo transfer. Clinical pregnancy was confirmed by the detection of a gestational sac with a foetal heartbeat by transvaginal ultrasound examination 14 days later.

### Statistical analysis

The statistical analysis was performed using SPSS version 13 statistical software. The continuous data were described as the mean ± sd and compared using the Mann–Whitney U rank sum test for two independent samples or the Wilcoxon signed ranks test for the paired samples. The rates were compared using the Chi square test and Fisher’s Exact Test when appropriate. The differences in the P-value <0.05 were considered to be statistically significant.

## Results

### Basal clinical characteristics

The ANA + group and the ANA- group had no significant differences in the age, duration of infertility, BMI, basal hormone levels, days of ovarian stimulation, total Gn dose, serum E2 level and endometrial thickness on the day of the HCG injection (Table 1).

**Table 1:** Basal characteristics in the ANA + group and the ANA- group

**Variables**	**ANA + group**	**ANA- group**	**P**
*Patients*	66	233	ns
*Age (yrs)*	32.33 +/- 4.25	31.41 +/- 3.84	ns
*BMI (kg/ m*^*2*^*)*	21.72 +/- 2.58	21.77 +/- 3.04	ns
*Duration of infertility (yrs)*	5.06 +/- 2.66	4.73 +/- 3.10	ns
*bFSH (IU/L)*	7.15 +/- 1.84	7.06 +/- 2.00	ns
*bLH (IU/L)*	4.61 +/- 2.85	4.41 +/- 2.16	ns
*bE2 (pg/ml)*	46.41 +/- 19.60	48.82 +/- 19.39	ns
*Days of ovarian stimulation*	12.21 +/- 2.36	12.65 +/- 2.18	ns
*Total Gn dose (U)*	2464.77 +/- 78.71	2399.25 +/- 803.25	ns
*E2 levels on HCG day (pg/ml)*	3014.28 +/- 1337.26	2952.86 +/- 1401.69	ns
*Endometrial thickness on HCG day (mm)*	10.46 +/- 1.64	10.96 +/- 2.04	ns

P < 0.05 was considered to be statistically significant.

The Titre **≤** 1:320 subgroup and the Titre > 1:320 subgroup also showed no significant differences in the age, duration of infertility, BMI, basal hormone levels, days of ovarian stimulation, total Gn dose, serum E2 level and endometrial thickness on the day of the HCG injection (Table 2).

**Table 2:** Basal characteristics in the titre ≤1:320 subgroup and the titre > 1:320 subgroup

**Variables**	**Titre ≤1:320**	**Titre > 1:320**	**P**
*Patients*	46	20	-
*Age (yrs)*	31.72 +/- 4.12	33.75 +/- 4.30	ns
*BMI (kg/ m*^*2*^*)*	21.96 +/- 2.55	21.16 +/- 2.63	ns
*Duration of infertility (yrs)*	4.98 +/- 2.65	5.20 +/- 2.75	ns
*bFSH (IU/L)*	7.00 +/- 2.02	7.49 +/- 1.33	ns
*bLH (IU/L)*	4.68 +/- 3.11	4.46 +/- 2.22	ns
*bE2 (pg/ml)*	45.97 +/- 21.09	47.43 +/- 16.12	ns
*Days of ovarian stimulation*	12.52 +/- 2.30	11.50 +/- 2.40	ns
*Total Gn dose (U)*	2553.0 +/- 838.50	2261.25 +/- 619.50	ns
*E2 levels on the HCG day (pg/ml)*	3017.61 +/- 1404.05	3006.64 +/- 1203.62	ns
*Endometrial thickness on HCG day (mm)*	10.40 +/- 1.74	10.60 +/- 1.42	ns

P < 0.05 was considered to be statistically significant.

The ANA + cycles without P + A and the ANA + cycles with P + A had similar days of ovarian stimulation, total Gn dose, serum E2 level and endometrial thickness on the day of the HCG injection (Table 3).

**Table 3:** Basal characteristics in the ANA + cycles without P + A and the ANA + cycles with P + A

**Variables**	**Without P + A**	**With P + A**	**P**
*Patients*	21	21	-
*Age (yrs)*	31.71 +/- 3.61	-	-
*BMI (kg/ m*^*2*^*)*	21.53 +/- 2.23	-	-
*Duration of infertility (yrs)*	4.62 +/- 2.44	-	-
*bFSH (IU/L)*	7.38 +/- 2.10	-	-
*bLH (IU/L)*	4.57 +/- 2.46	-	-
*bE2 (pg/ml)*	52.85 +/- 24.40	-	-
*Days of ovarian stimulation*	12.22 +/- 2.14	11.95 +/- 2.13	ns
*Total Gn dose (U)*	2719.64 +/- 808.35	2567.86 +/- 722.85	ns
*E2 levels on HCG day (pg/ml)*	2704.12 +/- 1305.23	3139.39 +/- 1417.05	ns
*Endometrial thickness on HCG day (mm)*	10.58+/- 1.93	10.59 +/- 2.59	ns

P < 0.05 was considered to be statistically significant.

### Fertilisation and embryo development in the ANA + group and the ANA- group

The proportion of cycles using the ICSI fertilisation program was similar in the ANA + and ANA- groups (57.6% vs. 54.5%, p = ns)*.* The semen parameters (i.e., the semen volume, sperm concentration and progressive motility) on the day of OPU were not significantly different in the ICSI cycles and IVF cycles, respectively, between the two groups.

The MII oocytes rate (78.1% vs. 82.6%) and the normal fertilisation rate in the ICSI/IVF cycles (70.5% vs. 83.1%, and 66.1% vs. 76.0%) in the ANA + group were significantly lower than those in the ANA- group, whereas the opposite case occurred for the abnormal fertilisation rate in the ICSI/IVF cycles (4.91% vs. 0.36%, and 4.33% vs. 2.33%). There were no significant differences between the groups in the cleavage rate and perfect and available embryo rates (Table 4).

**Table 4:** Fertilisation and embryo development in the ANA + group and the ANA- group

**Variables**	**ANA + group**	**ANA- group**	**P**
*OPU cycles (IVF + ICSI)*	66 (28 + 38)	233 (106 + 127)	-
*ET cycles*	52	184	ns
*Proportion of ICSI program (%)*	57.6 (38/66)	54.5 (127/233)	ns
*Semen volume (ml, ICSI cycles)*	3.46+/-0.74	3.96+/-0.86	ns
*Sperm concentration (×106/ml, ICSI cycles)*	16.86+/-13.34	15.45+/-11.23	ns
*Sperm progressive motility (%, ICSI cycles)*	11.97+/-9.74	10.96+/-8.44	ns
*Semen volume (ml, IVF cycles)*	3.67+/-0.87	3.86+/-0.84	ns
*Sperm concentration (×106/ml, IVF cycles)*	65.33+/-44.33	68.84+/-46.00	ns
*Sperm progressive motility (%, IVF cycles)*	40.28+/-16.78	35.23+/-15.84	ns
*Retrieved oocytes*	9.73 +/- 5.03	10.38 +/- 4.15	ns
*MII oocytes rate (%, ICSI cycle)*	78.1 (285/365)	82.6 (1112/1346)	0.047
*Normal fertilisation rate (%, ICSI cycle)*	70.5 (201/285)	83.1 (924/1112)	<0.001
*Abnormal fertilisation rate (%, ICSI cycle)*	4.91 (14/285)	0.36 (4/1112)	<0.001
*Normal fertilisation rate (%, IVF cycle)*	66.1 (183/277)	76.0 (815/1073)	<0.001
*Abnormal fertilisation rate (%, IVF cycle)*	4.33 (12/277)	2.33 (23/1073)	0.041
*Cleavage rate (%)*	97.7 (375/384)	98.5 (1713/1739)	ns
*Perfect embryo rate (%)*	63.7 (239/375)	66.5 (1139/1713)	ns
*Available embryo rate (%)*	78.7 (295/375)	82.9 (1420/1713)	ns
*Transferred embryos/cycle*	2.12 ± 0.55	2.11 ± 0.38	ns

P<0.05 was considered to be statistically significant.

**Note:** Sperm with progressive motility, including those that move fast in a straight line (grade a) and that move forward but tend to travel in a curved or bent motion (grade b). MII oocytes rate = M II oocytes/total oocytes in the ICSI cycle; normal fertilisation rate = 2PNs /total M II oocytes in the ICSI cycle or 2PNs/total oocytes in the IVF cycle; abnormal fertilisation rate = 1PNs + multi-PNs/total M II oocytes in the ICSI cycle or 1PNs + multi-PNs/total oocytes in the IVF cycle.

### IVF/ICSI outcomes in the ANA + group and the ANA- group

**Figure 1:**
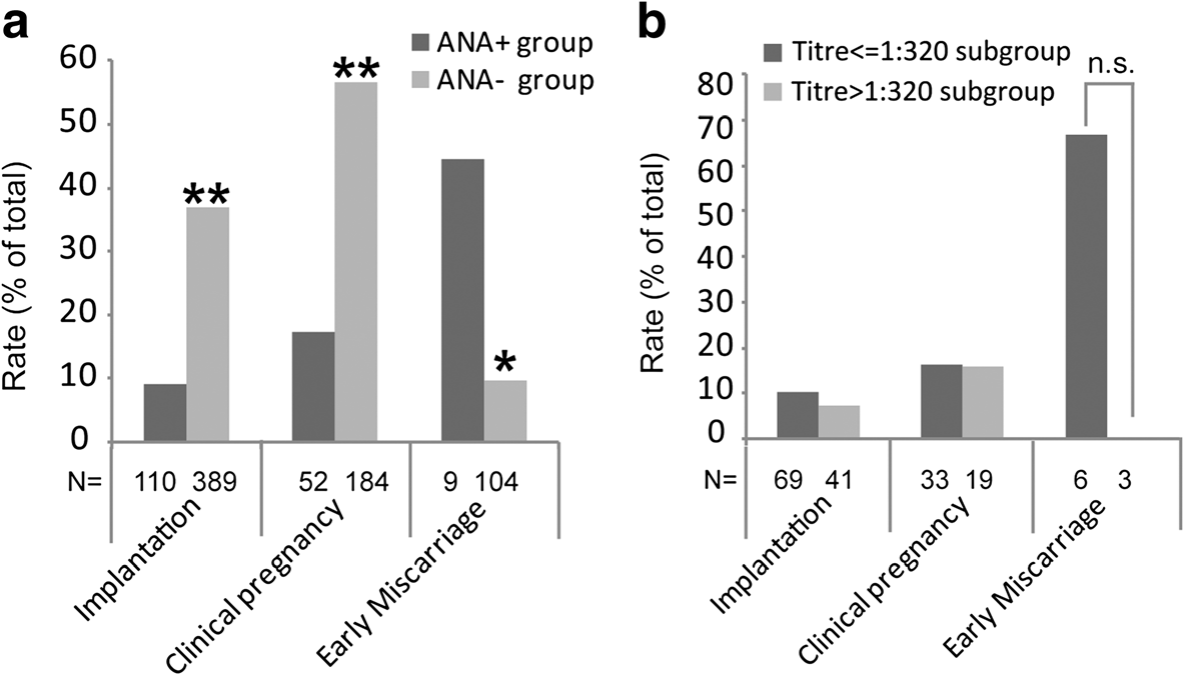
**Pregnancy outcome in IVF/ICSI cycles. (a)** The IVF/ICSI outcome in the ANA + group and the ANA-group. In the ANA + group, the implantation rate (9.09% vs. 36.8%) and the clinical pregnancy rate (17.3% vs. 56.5%) decreased significantly, and the early miscarriage rate (44.4% vs. 9.62%) increased markedly more than in the ANA- group. **(b)** The IVF/ICSI outcome in the Titre ≤1:320 subgroup and the Titre > 1:320 subgroup. No significant differences were found between the two subgroups in the implantation rate, the clinical pregnancy rate and the early miscarriage rate. * P < 0.05, ** P < 0.01.

The implantation rate (9.09% vs. 36.8%) and the clinical pregnancy rate (17.3% vs. 56.5%) in the ANA + group were significantly lower than those in the ANA- group, whereas the early miscarriage rate (44.4% vs. 9.62%) showed the opposite result (Figure 1-a).

### Fertilisation and embryo development in the titre ≤ 1:320 subgroup and the titre > 1:320 subgroup

The MII oocytes rate (73.6% vs. 80.4%) and the normal fertilisation rate in the ICSI/IVF cycle (64.1% vs. 73.6% and 57.6% vs. 68.3%) in the Titre > 1:320 subgroup were lower than those in the Titre **≤** 1:320 subgroup but without significance. The abnormal fertilisation rate in the IVF cycles (10.20% vs. 2.75%) in the Titre > 1:320 subgroup was higher than that in the Titre **≤** 1:320 subgroup. There were no significant differences between the subgroups in the cleavage rate and the perfect and available embryo rates (Table 5).

**Table 5:** Fertilisation and embryo development in the titre ≤ 1:320 subgroup and the titre > 1:320 subgroup

**Variables**	**Titre ≤ 1:320**	**Titre > 1:320**	**P**
*OPU cycles*	46	20	-
*ET cycles*	33	19	-
*Retrieved oocytes*	9.96 +/- 5.04	9.20 +/- 5.08	ns
*MII oocytes rate (%, ICSI cycle)*	80.4 (193/240)	73.6 (92/125)	ns
*Normal fertilisation rate (%, ICSI cycle)*	73.6 (142/193)	64.1 (59/92)	ns
*Abnormal fertilisation rate (%, ICSI cycle)*	5.18 (10/193)	4.35 (4/92)	ns
*Normal fertilisation rate (%, IVF cycle)*	68.3 (149/218)	57.6 (34/59)	ns
*Abnormal fertilisation rate (%, IVF cycle)*	2.75 (6/218)	10.2 (6/59)	0.034
*Cleavage rate (%)*	97.6 (284/291)	97.8 (91/93)	ns
*Perfect embryo rate (%)*	63.7 (181/284)	63.7 (58/91)	ns
*Available embryo rate (%)*	78.2 (222/284)	80.2 (73/91)	ns
*Transferred embryos/cycle*	2.09 +/- 0.46	2.16 +/- 0.69	ns

P < 0.05 was considered to be statistically significant.

### IVF/ICSI outcomes in the titre ≤ 1:320 subgroup and the Titre > 1:320 subgroup

No significant differences were observed between these subgroups in the implantation rate, clinical pregnancy rate and early miscarriage rate (Figure 1-b).

### Fertilisation, embryo development and outcomes in the ANA + cycles without P + A and the ANA + cycles with P + A

The semen parameters (i.e., the semen volume, sperm concentration and progressive motility) on the day of OPU were not different significantly in the ICSI cycles and the IVF cycles, respectively, between the ANA + cycles without P + A and the ANA + cycles with P + A.

After the prednisone plus low-dose aspirin adjuvant therapy, the number of 2PN (4.86+/-2.89 vs. 7.05+/-3.17), embryos (4.81+/-2.93 vs. 6.90+/-3.24), perfect embryos (3.24+/-2.41 vs. 4.76+/-2.93) and available embryos (3.62+/-2.22 vs. 5.76+/-3.25), as well as the pregnancy rate (12.5% vs. 57.1%) and the implantation rate (6.06% vs. 27.9%) increased significantly. The early miscarriage rate was not statistically analysed because of the small number of cases (Table 6).

**Table 6:** Fertilisation, embryo development and outcome in the ANA + cycles without P + A and the ANA + cycles with P + A

**Variables**	**Without P + A**	**With P + A**	**P**
*OPU cycles (IVF + ICSI)*	21(11 + 10)	21 (11 + 10)	-
*ET cycles*	16	21	-
*Semen volume (ml, ICSI cycles)*	3.54+/-0.81	3.09+/-0.87	ns
*Sperm concentration (×106/ml, ICSI cycles)*	14.64+/-11.21	16.34+/-10.65	ns
*Sperm progressive motility (%, ICSI cycles)*	12.87+/-10.97	15.34+/-11.56	ns
*Semen volume (ml, IVF cycles)*	3.44+/-0.93	3.64+/-0.83	ns
*Sperm concentration (×106/ml, IVF cycles)*	62.56+/-43.78	66.23+/-39.23	ns
*Sperm progressive motility (%, IVF cycles)*	46.89+/-15.45	43.89+/-17.68	ns
*Retrieved oocytes*	8.10 +/- 3.95	9.24 +/- 4.10	ns
*MII oocytes*	6.50 +/- 4.09	7.20 +/- 3.99	ns
*Two pro-nuclei*	4.86 +/- 2.89	7.05 +/- 3.17	0.004
*Perfect embryos*	3.24 +/- 2.41	4.76 +/- 2.93	0.037
*Available embryos*	3.62 +/- 2.22	5.76 +/- 3.25	0.008
*Embryos*	4.81 +/- 2.93	6.90 +/- 3.24	0.006
*Transferred embryos/cycle*	2.06 +/- 0.44	2.76 +/- 0.58	0.008
*Pregnancy rate (%)*	12.5 (2/16)	57.1% (12/21)	0.006
*Implantation rate (%)*	6.06 (2/33)	27.9 (16/58)	0.013
*Early miscarriage rate (%)*	100 (2/2)	25 (3/12)	-

P < 0.05 was considered to be statistically significant.

The mean number of embryos for transfer per cycle (2.76 +/- 0.58 vs. 2.06 +/- 0.44, P = 0.008) in the cycles with P + A was higher than that in the cycles without P + A (Table 6). The increased pregnancy rate in the cycles with P + A might be because of the medication effect and/or the greater number of embryos for transfer per cycle. It appeared that the pregnancy rate was not an appropriate indicator for evaluating the medication effect.

## Discussion

For several years, detection of autoantibodies has been recommended in clinical practice for women with infertility including the detection of ANA, APA and ATA. A link of APA with recurrent pregnancy loss has been established, and treatment based on anticoagulation such as subcutaneous heparin is effective [3]. As an organ-specific autoantibody, ATA affect pregnancy outcome negatively by damaging the thyroid function, and the thyroxin replacement therapy is efficacious in preventing foetal loss for patients with recurrent miscarriage [1,2]. However, the potential correlation between ANA and IVF/ICSI outcome and specific medication are less reported.

Previous reports showed that ANA were relevant to adverse reproductive events including recurrent spontaneous abortion, endometriosis, infertility, IVF failure and ovarian dysfunction [6-10]. One recent study proposed that ANA might impair oocyte quality and embryo development, leading to reduced pregnancy and implantation rates [10]. A further exploration [20] similarly suggested that IVF outcomes were markedly poorer in ANA + women, and this effect became worse with an increased level of serum ANA.

In this study, we found that the presence of ANA in serum predicted an adverse IVF/ICSI outcome, primarily reflecting the lower rates of MII oocytes and normal fertilisation and the reduced rates of implantation and clinical pregnancy, as well as the increased rates of abnormal fertilisation and early miscarriage. The cleavage rate and the perfect and available embryo rates were not significantly different between the ANA + women and the controls, which was a finding that did not coincide with that of Ying Y et al. [10]. We found that ANA-titre ≤1:320 women had as poor IVF/ICSI outcomes as the ANA-titre > 1:320 women, and only the abnormal fertilisation rate in the IVF cycles in the ANA-titre > 1:320 women was significantly higher. These results did not suggest the titre-dependent effect of ANA on IVF/ICSI outcomes, in agreement with Hasegawa et al. [21] and Taniguchi et al. [22].

In general terms, abnormal autoimmune conditions may impair all stages of fertility, leading to ovarian and testicular failure, implantation failure or pregnancy loss through different putative mechanisms [5]. It has been proposed that APA and A-β2-GPI could result in thrombosis of placental blood vessels, dysfunctions of trophoblasts in the peri-implantation period or an imbalance of maternal hormones [23-25]. Unlike APA and A-β2-GPI, the mechanism by which ANA determine reproductive failure remains speculative. Hasegawa et al. [21] held that ANA did not target specific organs and only presented as an abnormal degree of autoimmunity, based on their findings that the adverse IVF outcome was not ANA-titre dependent. One recent well designed study [26] demonstrated that the presence of APA, ANA or ATA in recipients using donor oocytes had no negative effect on pregnancy, implantation or miscarriage, which suggested that implantation failure might be because of poor-quality oocytes that may lead to a subsequent embryo development disorder. One *in vivo* test [27], in which anti-centromere antibodies were microinjected into mouse oocytes, showed the anti-centromere antibody could interfere with chromosome congression in the pro-metaphase. Shirota K et al. [18] considered that anti-centromere antibodies might infiltrate oocytes and lead to centromere dysfunction during meiosis and mitosis and impair the transition from MI to MII during oocyte maturation. Ying Y et al. [20] has confirmed that ANA exist in follicular fluid and embryos in ANA + patients, and serum and follicular fluid ANA negatively correlated with the number of high-quality embryos. The embryos co-cultured with IgG extracted from ANA + women were found to be severely impaired or even died [19].

Ando et al. [28] administered low-dose prednisolone (5 mg/d) or dexamethasone (0.5 mg/d) daily during the entire IVF cycle until the pregnancy test was performed in 51 lVF-ET cycles of patients positive for ANA, anti-DNA antibody, and/or lupus anti-coagulant (LAC), as well as 29 IVF-ET cycles of patients negative for any antibodies and discovered significant increases of pregnancy and implantation rates in the antibodies-positive patients with corticosteroid treatment but not in the antibody-negative patients. Hasegawa et al. [21] administered prednisolone (10 mg/d) plus low-dose aspirin (81 mg/d) to ANA + and/or APA + women from the first day of COH until pregnancy was confirmed by ultrasonography and discovered that the ANA + women with treatment had significantly better outcomes of IVF-ET (40.6% pregnancy rate and 20.3% implantation rate). Taniguchi et al. [22] administered prednisolone (15–60 mg/d) starting from the first day after OPU for 5 days in 56 IVF-ET cycles of 24 ANA + women and 167 IVF-ET cycles of 96 ANA-women and found the implantation rate and clinical pregnancy rate improved significantly in the ANA + woman but not in the ANA-women, which coincides with the findings of Ando et al. One previous prospective study administered prednisone plus aspirin for 4 weeks before IVF treatment to 52 women positive for ACA, ANA, anti-dsDNA antibody, rheumatoid factor, and/or LAC and ultimately obtained a satisfactory clinical pregnancy rate (32.7%) [29]. The present study pretreated ANA + women with prednisone (10 mg/d) plus low-dose aspirin (100 mg/d) (i.e., P + A) for three months before IVF treatment and observed that the ANA + cycles with P + A had markedly more 2PN, high-quality and available embryos, and an increased implantation rate. Our study also showed that the ANA titre was not relevant to the IVF/ICSI outcome and the adjuvant treatment was not closely related to the reduction of the serum ANA titre, which is in agreement with Hasegawa et al. [21]. In this study and that of Hasegawa et al. [21], women positive for autoantibodies were administered a 10-mg daily dose of prednisone or prednisolone, and this dosage is too low to reduce the autoantibody titres. Thus the corticosteroid effect may be derived from another mechanism such as an anti-inflammatory action or the regulation of immune cells as in the reduction of NK cells [21]. Low-dose aspirin for its anti-thrombotic effect may reduce uterine and intraovarian vascular resistance, improve blood perfusion and increase oocyte maturation, the high-quality embryos rate, and the implantation rate [21]. Although these five studies including the present work demonstrated a beneficial effect of corticosteroid or corticosteroid plus aspirin therapy, an ideal protocol for this adjuvant therapy (i.e., the indication of the patients, the drug selection and dosage, and the timing of commencement and end) requires further investigation.

In this study, we observed the detrimental effect of ANA on IVF/ICSI outcome and the beneficial effect of prednisone plus low-dose aspirin adjuvant treatment for ANA + patients. The comparison between the first IVF/ICSI cycle to a subsequent one would unavoidably yield bias, which is the limitation of our study. To minimise the bias, we rigorously selected the subjects, and there was a long interval between the first and the second IVF/ICSI cycles of at least 3 months. In our future work, we will attempt to perform a placebo-controlled, double blind, and prospective study.

## Conclusions

These observations suggest that ANA could exert a detrimental effect on the IVF/ICSI outcome that may not be titre-dependent, and prednisone plus low-dose aspirin adjuvant treatment could be useful in ANA + patients. This hypothesis should be verified in placebo-controlled, double blind, and prospective studies.

## Abbreviations

A: Aspirin; ACA: Anti-cardiolipin antibody; A-β2-GPI: Anti-beta(2)-glycoprotein I; ANA: Anti-nuclear antibody; APA: Anti-phospholipids antibody; ATA: Anti-thyroid antibody; anti-dsDNA antibody: Anti-double strand DNA antibody; COH: Controlled ovarian stimulation; ET: Embryo transfer; E2: Estradiol; Gn: Gonadotropin; GnRH: Gonadotropin-releasing hormone; HCG: Human chorionic gonadotropin; ICSI: Intracytoplasmic sperm injection; IFT: Indirect immunofluorescence method; IVF: In vitro fertilisation; LAC: Lupus anti-coagulant; OPU: Oocytes pick-up; P: Prednisone; STD: Sexually transmitted diseases; T3: Triiodothyrorine; T4: Thyroxine; TSH: Thyroid stimulating hormone; 2PN: Two pro-nuclei.

## Competing interests

The authors declare that they have no competing interests.

## Authors’ contributions

QZ and LW conducted the data collection, participated in the design of the study, and drafted the manuscript. BX and MHH participated in the processing of the data and the statistical analysis, and performed the diagramming. XHT and JJJ conducted the interpretation of the data and the revision for important intellectual content and grammar. YSL conceived of the study, participated in its design and coordination, and helped to draft the manuscript. All the authors read and approved the final manuscript.
